# A Preliminary Study on Productive Performance and Environmental Requirements of a Newly Established Breed: Nero di Lomellina Pig

**DOI:** 10.3390/ani15182655

**Published:** 2025-09-10

**Authors:** Annamaria Costa, Eleonora Buoio, Margherita Pallaoro, Edda Mainardi, Giorgio Mirra, Alessia Di Giancamillo, Silvia Michela Mazzola, Raffaella Rossi

**Affiliations:** 1 Department of Veterinary and Animal Sciences (DIVAS), University of Milan (UNIMI), Via dell’Università, 6, 26900 Lodi, LO, Italy; eleonora.buoio@unimi.it (E.B.); edda.mainardi@unimi.it (E.M.); silvia.mazzola@unimi.it (S.M.M.); raffaella.rossi@unimi.it (R.R.); 2 Department of Biomedical Sciences for Health (SCIBIS), University of Milan (UNIMI), Via Mangiagalli, 31, 20133 Milano, MI, Italy; margherita.pallaoro@unimi.it (M.P.); giorgio.mirra@unimi.it (G.M.); alessia.digiancamillo@unimi.it (A.D.G.)

**Keywords:** Nero di Lomellina pigs, traditional farm, thermoneutral-zone, heat stress, hair cortisol concentration, growth performances

## Abstract

The Nero di Lomellina (NL) pig, a newly established breed from the cross of the Poland China with the Berkshire breed, which was created to reconstitute a local breed called Nero di Cavour, was recognized by the Italian Ministry of Agriculture in 2020 (DM 12222/2020). The objective of this study is to investigate, for the first time, the zootechnical performance and environmental response of the NL breed in comparison with a commercial crossbreed (CH; Large White × Duroc). An evaluation was conducted of a traditional closed-cycle farming system rearing NL and CH mixed in the same units. Pigs were observed for two cycles of 10 months each, one with piglets born in winter and the other in summer, to evaluate the productive performance and stress response through hair cortisol (HCC) levels of the pigs according to environmental conditions. For this purpose, the temperature–humidity index (THI) was calculated for each phase of the two cycles, and the values were compared with the comfort zone values indicated by the Italian Classyfarm values. The NL pigs showed slower growth performance in the first phase, with a high THI (*p* < 0.05). In the R2 phase, the backfat thickness was higher in the NL than CH pigs (*p* < 0.001). The CH had consistently higher HCC compared with the NL pigs across the production phases, showing a potential higher response to chronic stress. It is worth noting that the NL weaners and growers seemed to better face a cold environment, referring to their growth performance in comparison with the commercial breed, although the local breeds usually show a slower growth rate and hair cortisol levels during the second cycle, which was characterized by prolonged low temperatures far from the comfort zones. More studies are needed to deepen the influence of the environment, as well as consider the outdoor conditions on the physiological status, productivity, and meat quality of this new breed.

## Simple Summary

This study investigates the productive parameters and stress resistance of the Nero di Lomellina pig breed, which was officially added to the Herdbook in 2020, compared with commercial hybrid pigs (Large White × Duroc) raised under the same conditions in a traditional farm located in the province of Pavia. The farm operates with traditional systems and lacks advanced microclimate control technologies. The aim of the present study is to define the thermoneutral zone of this newly established breed throughout two production cycles. Throughout the study period, environmental parameters such as temperature, humidity, and pollutant concentrations were continuously monitored. The pigs were weighed at the end of each production cycle and hair cortisol from 5% of the animals was examined to define the chronic stress status in the different phases. The Nero di Lomellina pigs showed a higher tolerance to climatic fluctuations, with a better resilience at low environmental temperatures, especially in comparison with the commercial crosses. The hair cortisol confirmed the higher adaptation capacity of these pigs in a barren environment. The lower critical temperature is the ambient temperature below which pigs must increase heat production to maintain a heat balance. The upper critical temperature is the ambient temperature above which pigs must increase their heat loss rate to achieve a heat balance.

## 1. Introduction

The standardization of pig production systems in intensive farming, which is characterized by the widespread use of commercial crosses, or hybrids, selected for efficiency and high performance, has led to a progressive reduction in the genetic biodiversity among breeding populations [1]. In the last decade, there has been growing interest in local and traditional pig breeds, not only for their role in biodiversity conservation but also for their rusticity, environmental adaptability, and potential to produce high-quality niche products [2,3]. This trend aligns with broader policy priorities, including those of the European Union’s Common Agricultural Policy, which encourages the protection and valorization of genetic resources in livestock systems [4].

Native pig breeds are typically better adapted to the agroclimatic conditions of their region of origin and exhibit greater resilience to environmental stressors, particularly in low-input or extensive farming systems. In contrast, commercial hybrids—selected primarily for their growth rate and feed efficiency in controlled environments—tend to have a narrower thermoneutral zone and greater susceptibility to heat stress [5]. Microclimatic parameters such as ambient temperature, relative humidity, and ventilation rate significantly affect animal welfare. When these conditions are suboptimal, physiological stress responses intensify, often leading to a decline in productive performances [6,7,8]. This highlights the importance of breed selection for environmental adaptability, especially in light of rising global temperatures and the growing demand for sustainable, resilient livestock systems. These traits make native breeds promising candidates for diversifying agri-food chains and improving the sustainability and robustness of pig farming under variable environmental conditions.

The Nero di Lomellina (NL) is a pig breed historically linked to the Lomellina area of Lombardy in Northern Italy. It shares phenotypic traits with the ancient Po Valley pig known as the Nero di Cavour [9]. During the 20th century, the breed underwent changes through systematic crossbreeding with breeds such as the Poland China and Berkshire to improve their performance and cold tolerance. While these practices enhanced their productivity, they also eroded the breed’s original genetic identity [10].

Although the current population is no longer genetically identical to its ancestral form, a phenotypic recovery and selection program was launched in the early 2000s based on animals maintained since the 1970s on a farm in the province of Pavia. With academic support, the program aimed to stabilize key morphological and productive traits. As a result, the NL pig was officially recognized by the Italian Ministry of Agriculture in 2020 as a reconstructed local pig population (Decree No. 12222/2020), with the aim of promoting the breeding of these native black pigs in order to improve biodiversity conservation.

Despite this official recognition, no studies have yet investigated the productive parameters and the environmental adaptability of this population. Some data on muscle development of NL and commercial hybrid (CH; Large White × Duroc) pigs have recently been reported by Pallaoro et al., 2025 [11].

The present research represents the first effort to characterize the productive performance and environmental resilience of the NL breed within a traditional closed-cycle farming system. The study compares this breed with CH, whose performance, stress response, and thermoregulatory range are already well documented [5,6,7,8].

To better understand the animals’ physiological adaptability to seasonal variations, this preliminary study was assessed across two productive 10-month rearing cycles, the first with piglets born in winter, and the other with piglets born in summer.

## 2. Materials and Methods

### 2.1. Farm and Animals

The study was conducted on a commercial farm located in Northern Italy (Azienda Agricola S. Michele s.r.l, Vigevano, PV, Italy). The farm operated a closed-cycle production system, and two pig breeds were raised in the farm: purebreed NL and CH. The farm housed approximately 2000 pigs of NL breed, and 400 pigs of CH breed reared up to slaughter weight. The animals were fed with corn-based commercial diets formulated to meet the requirements for all nutrients in the different breeding phases [12].

The farm operated under traditional–conventional conditions, where the buildings were not insulated and not equipped with mechanical ventilation, heating, or cooling systems, and the air volume exchanges were naturally provided by windows and the windshield.

#### 2.1.1. Farrowing Unit

**Figure 1:**
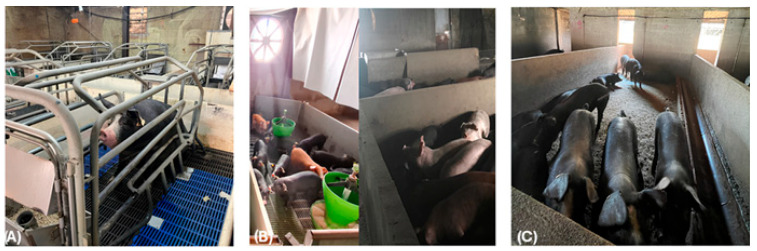
(**A**) Farrowing room, (**B**) post-weaning and growing unit, and (**C**) fattening unit.

The sows entered the farrowing unit one week before delivery and were moved to the heat observation unit when the piglets were weaned at 28 days of age (Figure 1A).

The farrowing unit was located within a dedicated building (11.75 × 13.4 × 3.8 m) equipped with six windows (three per long side), each measuring 80 × 78 cm. The room contained 21 traditional farrowing pens arranged in three rows. The dimensions of the pen were as follows:

- Farrowing pen: 2.78 × 1.64 m;
- Farrowing crate: 2.00 × 0.65 m;
- Feeder trough: 0.50 × 0.43 m.

The infrared lamps above the designated creep areas, in conjunction with electric heaters in the first days after birth, contributed to the heating of the nest zone. The flooring was fully slatted, allowing manure to drop into an underlying deep pit that was managed via overflow. In the following cycles, the pigs were reared together, as usual, on the farm.

#### 2.1.2. Post-Weaning and Growing Units

The post-weaning phase was organized in two distinct sectors, where the first unit was organized as an open space: the first room lodged piglets that were moved from the farrowing room to post-weaning, while the second room allowed for piglet acclimatization from the growth phase to the fattening phase (Figure 1B).

Fresh air was provided through high-positioned windows on the internal wall facing the main corridor, while the exhaust air was extracted by two fans on the external wall. The floor consisted of slatted floor over a deep pit system. Dry feed was administered to piglets through bell-shaped troughs.

After approximately one month in the post-weaning room, at 20–25 kg of live weight, the pigs were transferred to a growing room for a period of two months to reach 50 kg of live weight. The growing room unit was like the future fattening area of the farm: a barren environment with two rows of 3 batches (3 × 4 m) separated by a 1 m alley, the floor was full concrete, and the unit was naturally ventilated via windows that provided fresh air. Feed was manually administered by linear feeders.

#### 2.1.3. Fattening Unit

The fattening unit was a single building organized into batches of identical dimensions (3 × 4 m) arranged in two rows (Figure 1C). A central 70 cm wide alley separated the two rows, facilitating animal inspection, handling, and the use of a mobile scale for animal weighing. Each batch was equipped with a full concrete floor, and it was connected to an external dunging area with a slatted floor. The windows and openings to external paddocks facilitated the circulation of fresh air within the structure, allowing air volumes to exchange through a continuous ridge on the roof top. This configuration enabled natural ventilation in the piggery.

### 2.2. Monitoring Seasons

Two rearing cycles were observed in the present study: the first cycle involved pigs born in December 2021 and slaughtered in October 2022 (R1); the second one included animal born in September 2022 and slaughtered in July 2023 (R2). Both cycles, see Table 1, lasted about 10 months. The variables assessed throughout the study included both environmental parameters and animal-based indicators, including the productive performance and stress status.

#### 2.2.1. Animals Productive Performances

For each cycle, 5% of the NL and CH male pigs (*n* = 14 per breed) were randomly selected from the litters at birth, individually marked for identification, and monitored from birth to slaughter as a representative subset of their respective populations, totaling around 300 animals. The individual body weight of the pig at birth (T0) and in the weaning (T1), growing (T2), and fattening–finishing phases (T3) were recorded. At the same time, hair sampling was performed for cortisol detection. The backfat thickness at P2 (measured 6.5 cm from the midline over the last rib) was assessed in the growing phase and at slaughter using an ultrasound scanner (Wifi SU-2; TECNOVET S.L., Centelles, Spain).

#### 2.2.2. Environmental Monitoring

A detailed environmental monitoring protocol was carried out to characterize the microclimatic conditions and air quality within the housing facilities across the two rearing cycles.

Key microclimatic parameters, such as air temperature, relative humidity, and dew point, were continuously monitored with a frequency interval of 15 min by portable data loggers (PCE-HT 71N), which were placed in seven different locations within each room for the assessment of temperature and relative humidity fluctuations throughout the study period.

The air quality was assessed twice a week using a photoacoustic gas analyzer (BK INNOVA 1512, Lumasense Technologies, Raunheim, Germany) to determine concentrations of ammonia (NH_3_), carbon dioxide (CO_2_), methane (CH_4_), and nitrous oxide (N_2_O) in the different rearing cycles in 5 different points in the rooms to identify potential negative effects of pollutants on the animals in each phase.

The integration of data from continuous monitoring allowed for the characterization of the microclimate conditions in the different housing units during both rearing cycles.

##### Thermal Environmental Classification for the THI and the Thermoneutral Zone for the Nero di Lomellina Pig

Environmental monitoring data collected during the two production cycles were used to evaluate and compare the microclimatic conditions experienced by the pigs in the different growing phases. Data were then compared with the comfort temperatures for the various categories of pigs indicated by the Italian Classyfarm [13] and with reference thresholds reported in the literature to assess the compliance with optimal welfare standards for hybrid breeds.

The data were analyzed and graphically plotted, overlapping the heat stress and the thermoneutral zone based on the data provided by Zimmerman et al. [14], as considered by the Italian Classyfarm for optimal environmental conditions in piggeries, suggesting comfort temperatures for each weight category range. The indications are 24–29 °C for 5–14 kg, 21–27 °C for 14–23 kg, 16–21 °C for 23–34 kg, 13–21 °C for 34–82 kg, and 10–21 °C for 82 kg and above (finishers and breeders). The relative humidity, as a reference, is ideally between 50 and 75% [15]. Regarding piglets in the farrowing room, the recommended temperatures are 32 °C at the beginning of the cycle (delivery day) and around 24 °C at the third week of age (around 5 kg of live weight (LW) [16]).

It must be stressed that the inside temperatures in piggeries require adjustments according to the ventilation rate; air velocity; type of floor; and above all, the insulation level of the building [17].

To assess the potential thermal stress, the temperature–humidity index (THI) was considered as the best indicator for the climatic conditions. The THI was calculated according to [18,19,20,21]

(1) $$THI=0.8\times T+RH\times \left(T-14.4\right)+46.4$$

where

T is the environmental temperature (°C);
RH is the relative humidity (%) as a proportion.

Table 2 reports the data about climatic requirements of pigs [13,14,15]; in the last column, the corresponding THIs are shown.

### 2.3. Hair Cortisol Concentration

#### 2.3.1. Sampling Procedure

The hair sampling from the NL and CH pigs was carried out at different stages: birth (T0—piglets that had died due to crushing), weaning phase (T1—at about 8 kg LW), growing phase (T2—at about 60 kg of LW), and at the end of the fattening period (T3—at about 170 kg). The same weighed animals were processed for hair analysis (*n* = 14 per breed and cycle). The number of animals sampled was chosen according to Costa et al. [22].

**Figure 2:**
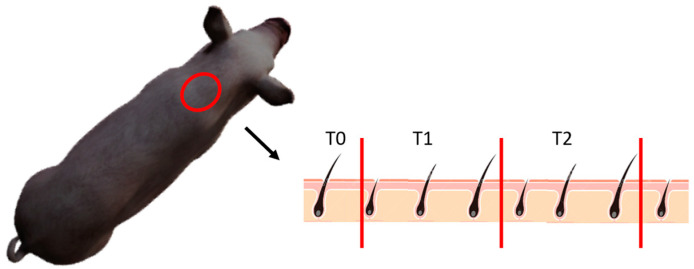
In the red circle, the area shaved for cortisol detection.

Hair sampling was performed by shaving an area approximately 10 × 10 cm between the neck and shoulders [23] using a double-blade razor, ensuring the hair was clipped as close as possible to the skin to collect the total hair length. This method minimized the inclusion of hair follicles, which could contain cortisol via blood contamination or due to potential endocrine activity [24,25]. The neck region was consistently selected across all the samples to avoid concentration variability between different body areas and to minimize potential contamination from fecal cortisol [26,27] (Figure 2). All the collected hair samples were stored in airtight nylon bags and kept in a cool, dry environment until analysis [28].

#### 2.3.2. Cortisol Extraction Protocol

The cortisol extraction from hair samples followed a previously validated protocol [23]. Briefly, 20 mg of clean, dry hair was weighed into sterile conical-bottom tubes (Sarstedt, Milan, Italy). Each sample received 1.2 mL of 99.9% pure methanol (Sigma-Aldrich, Milan, Italy). The tubes were sealed and sonicated for 30 min (Branson 2510, Branson Ultrasonic Corp, Brookfield, CT, USA). The samples were incubated for 12 h in a shaking water bath (100 rpm) at 50 °C (Grant OLS200, Fisher Scientific, Loughborough, UK). After the incubation, the samples were centrifuged at 3500 rpm for 15 min at 4 °C. A 1 mL aliquot of supernatant was transferred to 2.5 mL Eppendorf tubes and evaporated under a fume hood using a stream of ultrapure nitrogen at 45 °C (CentriVap, Labconco, Kansas City, MO, USA).

The dry residues were reconstituted in 1 mL of Phosphate Buffered Saline (PBS, Merck, San Jose, CA, USA) and immediately used for analysis. Cortisol concentrations were determined using a commercial enzyme immunoassay validated for porcine biological matrices (Porcine Cortisol ELISA Kit Competitive, BT BEA0011, Yangpu Dist, Shanghai, China). The samples were aliquoted into microplate wells in duplicate (50 µL per well), following the manufacturer’s protocol. Absorbance was read using a microplate reader (Multiskan EX, LabSystem, Thermo Fisher Scientific, Milan, Italy) at 450 nm. The cortisol concentrations (expressed in pg/mg) were calculated using a standard curve generated with dedicated ELISA analysis software v.1.0 (Gain Data ELISA Calculator, 2025).

### 2.4. Statistical Analysis

Environmental data were analyzed to determine the mean, maximum, and minimum T and RH values recorded during the study to climatically characterize the housing facilities and to identify the thermoneutral zone (TNZ) for the breeds under investigation.

The temperature–humidity index (THI) was calculated to assess the risk conditions of thermal stress experienced by the animals, considering the large daily temperature fluctuations typical of the uninsulated and unconditioned buildings in the study area.

The data on the growth performance and HCC were analyzed using a generalized linear model (GLM) to evaluate the differences between the breeds (CH and NL) and across the two observed rearing cycles (R1 and R2). The analysis included the different growth phases to identify potential interactions between the breeds and phases.

All statistical analyses were performed using SAS 9.4 software (SAS Institute Inc., Cary, NC, USA). The results were considered statistically significant for *p* < 0.05.

The weight data were further used to model the growth curve of each pig using a modified Gompertz model (Proc NLIN, Gauss method, SAS 9.4), which was selected for its ability to flexibly represent the asymptotic growth dynamics typical of livestock animals and other biological processes.

The modified Gompertz model [29,30] is described by the following equation:

Model (LW)= (a)(b) e(tlnc)

where

- (a)(b) represents the weight estimate at time 0 (birth weight);
- (a) represents the asymptotic weight;
- (c) is a parameter indicating the growth rate over time;
- t is the time considered in the model (days, 0–500).

The data were processed using Proc N-Lin of SAS 9.4 software (SAS Institute Inc., Cary, NC, USA) to produce non-linear regressions.

## 3. Results

### 3.1. Animal Growth Performance

In Table 3, the average body weight recorded for both breeds (NL and CH) are reported for each production phase and production cycle (R1 and R2), along with the backfat thickness in the growing and finishing phases.

At birth, no difference was observed for piglets’ weight from the NL and CH piglets within the production cycles. Even if the piglets were heavier in the second cycle (*p* < 0.001), this data did not influence the growth performances between breeds during this cycle.

At weaning, the CH pigs were significantly heavier than the NL pigs in R1 (*p* < 0.05), while no difference was found for R2. This trend was reflected in the average daily gain during the lactation phase (ADG), where the NL pigs in R1 demonstrated a slightly higher gain (0.288 ± 0.027 kg/day) compared with the CH pigs (0.248 ± 0.023 kg/day). In R2, the differences in the ADG between the breeds were not significant (NL 0.294 ± 0.059 kg/day; CH 0.274 ± 0.032 kg/day).

In the growing phase, the CH pigs in R1 continued to display higher body weights (*p* < 0.05), which was reflected in significantly higher ADG values influenced by season (*p* < 0.05). In R1, the fattening NL pigs showed the highest ADG (0.546 ± 0.059 kg/day), while the CH pigs in R2 exhibited the lowest growth rate in this phase (0.445 ± 0.099 kg/day). In the R2 production cycle, the weight of the pigs was lower (61.9 ± 1.5 kg vs. 55.9 ± 1.4 kg; *p* < 0.05) than in the R1 cycle.

At the end of the R1 fattening phase, the weight of the CH pigs was higher than the NL (179 ± 2.7 kg vs. 174 ± 2.7 kg; *p* < 0.05), supported by ADG values showing a strong seasonal effect (*p* < 0.001). The CH pigs in R1 achieved the highest ADG (0.691 ± 0.061 kg/day), while the NL pigs in R2 had lower gains (0.617 ± 0.034 kg/day).

In the R2 production cycle, the weight of the pigs was lower (176.9 ± 2.4 kg vs. 169.3 ± 1.2 kg; *p* < 0.05) than in the R1 cycle.

Overall, the total average daily gain was significantly affected by the season (*p* < 0.001), with the highest values recorded in R1 for both breeds (NL: 0.598 ± 0.016 kg/day; CH: 0.579 ± 0.039 kg/day) and lower values in R2 (NL: 0.535 ± 0.016 kg/day; CH: 0.548 ± 0.011 kg/day).

The backfat thickness was affected by the breed only in the R2 phase, with a higher backfat thickness in the NL than CH (*p* < 0.001). An overall difference was also observed between the breeding phases (17.5 mm vs. 20.6 mm; *p* < 0.001). This may be related to the high environmental temperature in the last month of fattening in the R1 cycle.

**Figure 3:**
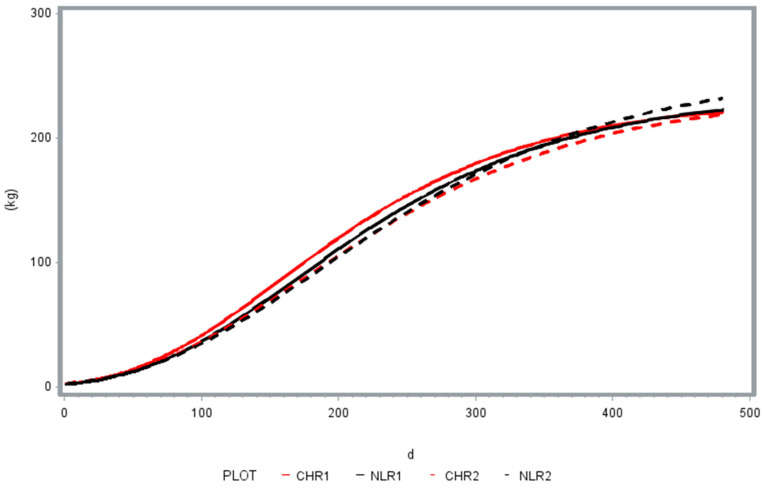
Growth curves for the LW and NL pigs in the two cycles.

Table 4 shows the parameters a, b, and c for the Gompertz curve, with the related SEM, and Figure 3 shows the shapes of the growth curves of the NL and CH groups in the two phases up to 500 d of age for LW prediction purposes. The growth trends of the groups were well defined by the Gompertz curve, with R^2^ ranging from 0.995 to 0.998.

Parameter a, indicating the asymptotic weight of pigs, was significantly higher for the NL in the second cycle of observation (*p* < 0.01). c, the parameter indicating the growth rate, was higher for the NL in the second round (*p* < 0.05). As shown by the graphic in Figure 3, the NL group showed a higher potential LW at 500 d of age in the second cycle.

### 3.2. Monitoring Survey for Air Quality in the Pig Houses

The air quality parameters are summarized in Table 5, reporting average concentrations of major pollutants. Hydrogen sulfide (H_2_S) was consistently below the detection limits; therefore, it is not reported. It is evident that there were high mean ammonia concentrations measured in the farrowing room during the first cycle due to the scarce ventilation rate, which is typical of winter on traditional pig farms.

### 3.3. Environmental Data

#### 3.3.1. Thermal Environmental Classification: Thermoneutral Zone and THI

**Figure 4:**
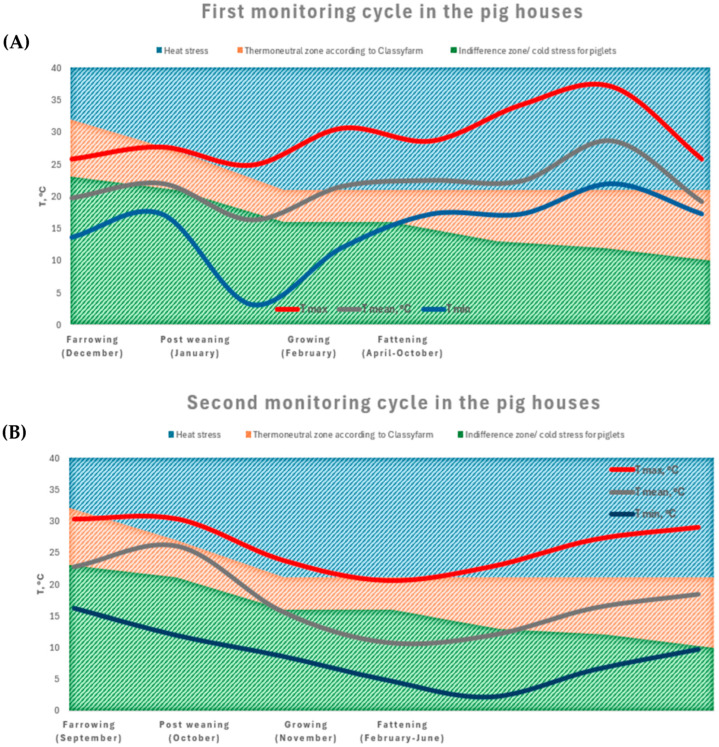
Temperatures (maximum and minimum values) trends recorded in the different rooms during the two rearing cycles, R1—(**A**) and R2—(**B**), referring to the comfort zone of pigs according to the Italian Classyfarm recommendation.

Figure 4A,B present the temperatures (maximum, mean and minimum values, red and blue lines) during each rearing unit in the two-rearing cycles (R1 and R2). These climatic areas overlap with a graphic describing the heat stress and the comfort zone, included in the thermoneutral zone, which is better discussed in the THI calculation section.

These data highlight a strong influence of seasonal variations on the thermal environment of the farm, with barren non-insulated rearing rooms and indoor temperatures often out of the desired ranges.

In R1, during winter, the temperatures in the farrowing and post-weaning units often failed to meet recommended values and thresholds, while in R2, the indoor conditions more closely reflected external temperature trends. The fattening unit showed high thermal variability characterized by overheating, especially in the first cycle.

##### The Farrowing Phase

The recommended thermal conditions for piglets typically range from approximately 32 °C on day one post-farrowing to 26 °C at weaning, as per established thermoneutral zones for neonatal swine [17]. In the present study, however, the farrowing room temperatures during cycle R1 dropped as low as 13.1 °C (T min), with a maximum of 26 °C—well below the thermoneutral threshold of 30 °C. In contrast, cycle R2 showed more favorable conditions, with temperatures closer to the recommended range (Tmin = 17 °C; Tmax = 30 °C), although still at the lower limit during early life stages. Such suboptimal thermal environments may increase cold stress and energy expenditure for thermoregulation, potentially affecting growth trajectories and survival rates in early life.

##### The Post-Weaning Phase

During the post-weaning phase, the ambient temperatures ranged from 13 °C to 26 °C across both production cycles. The functional thermoneutral zone for weaned piglets appears to span approximately 17 °C to 28 °C. Consequently, although environmental conditions during this phase were largely within the classical TNZ definitions, they may have imposed mild cold stress, particularly during the R2 cooler months.

##### The Growing Phase

During the growing phase, the temperatures ranged from 2 °C to 25 °C, in the first cycle, and during the second cycle, the microclimatic conditions ranged from 2 °C to 25 °C.

##### The Fattening Phase

In the fattening phase, optimal temperatures for maximizing feed efficiency and minimizing thermal stress are generally considered to lie between 15 °C and 18 °C [7]. However, as shown by Figure 4, both the R1 and R2 cycles recorded frequent and sustained exceedances of this range, especially during late spring and summer months: the maximum temperatures reached 38 °C in June (R1) and 29 °C in July (R2), well above the upper critical temperature for finishing pigs (24 °C). Such thermal loads are expected to impair performance by reducing the voluntary feed intake, elevating the maintenance energy requirements, and altering the endocrine responses.

##### The THI Definition: Thermoneutrality, Comfort, and Discomfort Zones

THI data from both cycles further illustrates the thermal stress burden (Table 6). In the farrowing units, the conditions remained largely thermoneutral in both cycles, with R2 showing occasional peaks (THI max = 81), which is still within tolerable limits.

The minimum values of the THI were lower with respect to the desired minimum threshold (62) as a description of the whole room, and the presence of IR lamps compensated for the piglets’ thermal requirements in the creep areas.

In the post-weaning phase, R2 showed higher mean values, potentially leading to thermal stress. While R1 stayed within the thermoneutral limits, R2 reached a THI of 82—indicative of moderate heat stress.

During the growing phase, both cycles exhibited substantial thermal variability due to poor insulation. The maximum THI values indicated moderate heat stress in both cases, which was more pronounced in R1, especially during the end of the cycle.

**Figure 5:**
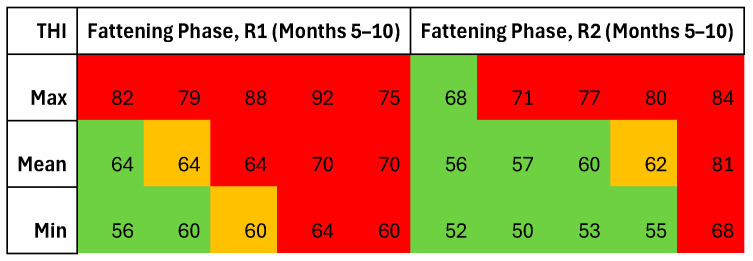
Thermoneutral classification based on THI thresholds—comfort (green), mild heat stress (yellow), moderate (orange), and severe (red) during the fattening phase in the two cycles.

In the fattening phase (see Figure 5), regarding the heat stress, R1 showed a gradual shift from moderate to severe heat stress, with the THI peaks > 90 in the summer months. R2 followed a similar pattern but exhibited delayed and slightly lower stress peaks—except when the cycle ended, in month 10, where R2 exceeded R1 (THI max: 92).

Overall, R1 was associated with more prolonged exposure to high THI values during the final rearing stages, while R2 showed shorter but intense heat episodes, particularly during weaning and the late fattening period. The trend of the THI in the two cycles is reported in Figure 5.

### 3.4. Hair Cortisol Concentration

**Figure 6:**
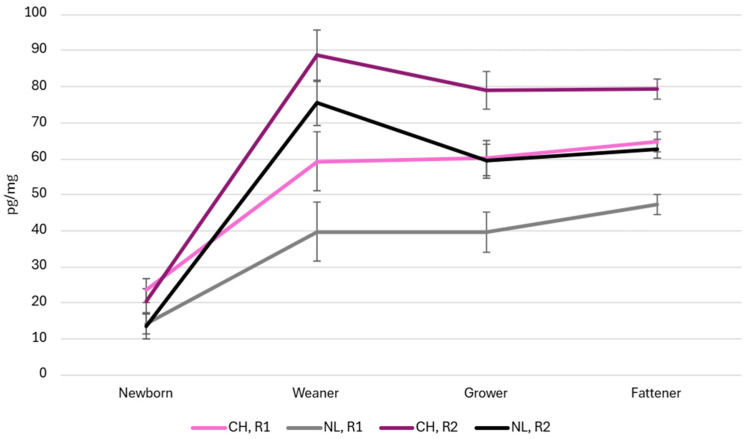
Hair cortisol concentration for the CH from R1 cycle (pink line), NL from R1 cycle (grey line), CH from R2 cycle (lilac line), and NL from R1 cycle (black line) at the different sampling points during the two production cycles (R1 and R2).

The data of hair cortisol concentration in the CH and NL are reported in Figure 6, subdivided according to the observed cycle R1 and R2 and the different sampling points.

The newborn cortisol concentrations were similar between the breeds and across cycles, except for the difference between the CH and NL in the R1 cycle (23.4 ± 3.3 pg/mg vs. 14.1 ± 2.8 pg/mg; *p* < 0.05).

At weaning, the cortisol levels increased markedly in both cycles and both breeds. In the CH pigs, the cortisol levels increased (*p* < 0.05) from 59.3 ± 8.2 pg/mg in the R1 cycle to 88.7 ± 7.2 pg/mg in the R2 cycle. In the NL pigs, the cortisol concentrations increased *(p* < 0.001) from 39.7 ± 0.57 pg/mg (R1) to 71.5 ± 0.44 pg/mg (R2). The CH pigs consistently showed higher cortisol levels than the NL pigs within each cycle and inter-cycle, indicating a more pronounced stress response.

In the growing phase, the CH pigs showed a higher HCC value than the NL in both cycles (*p* < 0.001). In the R1 cycle, the HCC value was 60.2 ± 4.84 pg/mg in the CH and 39.5 ± 5.59 pg/mg in the NL pigs (*p* < 0.001). In the R2 cycle, the HCC value was 78.9 ± 5.18 pg/mg in the CH and 59.3 ± 4.57 pg/mg in the NL pigs (*p* < 0.001). In the R2 cycle, the cortisol levels increased markedly more (*p* < 0.05) than the first one.

At the end of the fattening phase, the cortisol levels increased markedly in both cycles and both breeds. The HCC levels increased markedly more in the R2 cycle (*p* < 0.01) than the first one. In the CH pigs, the cortisol levels increased (*p* < 0.001) from 64.7 ± 2.80 pg/mg in the R1 cycle to 79.4 ± 2.80 pg/mg in the R2 cycle. In the NL pigs, the cortisol concentrations increased (*p* < 0.001) from 47.4 ± 2.80 pg/mg (R1) to 62.6 ± 2.59 pg/mg (R2).

## 4. Discussion

This study evaluated the growth performance and physiological stress responses of Nero di Lomellina reared in a traditional piggery together with commercial crosses while monitoring the microclimatic conditions, with the aim to define their climatic requirements.

Although the low weight at birth in the first rearing cycle, piglets reached a good live weight at weaning for both breeds. The NL showed a significantly lower live weight in comparison with the CH pigs in all production phases of the R1 cycle. In the R1 cycle, during winter, the temperatures in the farrowing and post-weaning units often failed to meet recommended and threshold values and were characterized by more prolonged exposure to high THI values during the final rearing stages. In the R2 cycle, even if the birth weight was higher than in the R1 cycle, no difference in the growth performances was observed between the NL and CH breeds. In this cycle, shorter but intense heat episodes, particularly during weaning and the late fattening period, were observed. This data is also evident when considering the lower backfat thickness recorded in the R1 cycle compared with the R2 cycle, which was related to heat stress. In this condition, a decrease in pig backfat thickness occurs because the animals reduce their feed intake in order to lower their metabolic heat production.

The parameters of the modified Gompertz curve showed a better productive performance of the NL group in the second cycle, and a higher potential mature weight for pigs slaughtered as heavy fatteners at 550 d of age. This can be explained by the fact that generally, local pig breeds, such as Nero di Lomellina, derived from crosses of Berkshire and Poland China pigs, show low muscular development and high potential for fat deposition.

The environmental analysis revealed acceptable pollutant concentrations throughout the different phases, except for a high concentration of ammonia in the farrowing room in the first cycle. According to Buoio et al. [16], recommended thresholds for maintaining good air quality in pig housing are <10 ppm for ammonia (NH_3_) and <3000 ppm for carbon dioxide (CO_2_). In R1, during the winter months, both thresholds were exceeded in the farrowing unit (NH_3_: 13.04 mg/m^3^; CO_2_: 4191 mg/m^3^), likely due to reduced ventilation and consequent gas accumulation [31]. Elevated CH_4_ and N_2_O concentrations observed in these conditions may reflect increased microbial fermentation activity in manure under low-ventilation environments [32]. In contrast, pollutant levels were generally lower in the fattening units across both cycles, possibly due to the use of natural ventilation systems.

Significant seasonal differences in environmental conditions were observed across the production phases. In R1, the farrowing period was marked by lower temperatures and higher humidity, with the NH_3_ and CO_2_ concentrations exceeding recommended welfare thresholds. This was likely due to the limited winter ventilation [31,32,33]. In contrast, the R2 cycle showed proper air exchange, which reduced the gas accumulation and resulted in more favorable conditions for the piglets.

Similar trends were observed during the post-weaning phase, where both rearing cycles showed suboptimal temperatures. However, greater variability and lower minimum temperatures in R1 suggest more overall challenging environmental conditions.

In the fattening phase, the temperatures frequently exceeded the TNZ in both cycles, particularly in R1, which caused a more prolonged exposure to heat stress. These data highlight that the NL fattening pigs, for example, have a wider range of the TNZ in comparison with the CH, determining a higher upper critical temperature up to a mean of 28 °C.

The TNZ is fundamental for assessing how pigs respond to thermal environments. It defines the ambient temperature range within which animals can maintain homeostasis without increasing metabolic effort [34]. Pigs are particularly susceptible to heat stress due to their limited thermoregulatory capacity, leading to a reduced feed intake, altered metabolism, and lower growth rates when exposed to temperatures above the TNZ [35,36].

Although most literature focuses on heat stress, given its widespread impact on intensive pig farming, it is important to recognize that cold discomfort can also significantly affect welfare, especially in younger pigs. Due to their lower body mass and immature thermoregulatory systems, piglets and weaners are especially vulnerable to cold. One of their main adaptive strategies is huddling, which reduces the exposed surface area, minimizes heat loss energy expenditure, and maintains their body temperature [37].

While less discussed than heat stress, cold exposure can still have substantial implications for animal welfare, particularly in early life stages. In the present study, the mortality rates exceeded 10% during the early growth phases. This elevated mortality was closely linked to the low temperatures recorded within the housing facilities, above all in R1, and in general to a high crushing rate by sows in the crates. In fact, piglets in this condition are more susceptible to cold stress, malnutrition, and physical injury from their mother due to their immature thermoregulatory abilities, limited energy reserves, and reduced vitality [38]. Indeed, experimental evidence supports the hypothesis that prolonged exposure to low temperatures can impair immune function [39].

It is worth noting that NL weaners and growers seemed to better face cold environment, referring to their better growth performance, although the local breeds usually show slower growth rate, and hair cortisol levels during the second cycle, characterized by prolonged low temperatures, far from the comfort zones.

It is worth noting that the NL weaners and growers seemed to better face the cold environment, referring to their growth performance comparable with the commercial breed (although the local breeds usually show a slower growth rate), and hair cortisol levels during the second cycle, which was characterized by prolonged low temperatures far from the comfort zones with a moderate THI.

This study incorporated lower temperature thresholds to identify conditions of cold exposure, even when these did not necessarily constitute cold stress per se, but rather a deviation from TNZ that could compromise comfort—especially in early growth stages.

It has to be stressed that while temperature remains the primary indicator of heat stress in pigs [34], relative humidity (RH) also played a significant role by limiting heat dissipation via respiration. The temperature–humidity index (THI), although more commonly used in cattle, offers a useful integrated measure of thermal discomfort in pigs, especially where evaporative cooling is limited. In this study, the THI values indicate exposure to heat stress in both cycles, particularly during the fattening phase of R1, where the elevated THI levels persisted over time, with critical THI values up to 92 at the 9th month of age. At this THI value, the authors found a low voluntary feed intake, higher breathing rate, and higher skin temperature, which in non-sweating animals like pigs represent a combination of factors indicating heat stress [19]. During the second rearing phase, with an extremely high THI up to 84, the NL pigs showed a better backfat deposition in comparison with the CH pigs. The use of the THI, a parameter widely used to prevent heat stress in dairy cattle, is relatively new in pig production and could be a useful tool to better define the TNZ of local breeds of pigs [19] and for pigs reared in traditional piggeries in these times of noticeable climatic changes.

HCC has emerged as a reliable biomarker of chronic physiological stress in pigs, integrating cumulative exposure to various stressors [40,41]. While not specific to heat stress alone, HCC reflects the animal’s overall adaptive response to persistent challenges—including nutritional deficits, metabolic strain, social interactions, and environmental factors such as air quality [16], and it represents a non-invasive chronic stress marker [24].

In the present study, the mean HCC levels reveal distinct patterns across genotypes and production cycles. Notably, pigs from the second cycle (R2) showed systematically higher cortisol concentrations than those from R1 at all time points, except T0.

Samples collected from piglets after delivery showed a difference between the CH and NL breeds in the first rearing cycle, with lower values for NL. The modest values of HCC in newborns seem to be related to limited in utero cortisol accumulation and minimal hair development in newborns [42]. This supports the hypothesis of maternal programming: environmental conditions experienced by the sow during gestation can influence the stress phenotype of piglets [43].

Moreover, the elevated HCC levels observed in both breeding phases in the NL and CH pigs may be explained by the stress associated with weaning, as previous data showed that change in social, environmental, nutritional, and structural factors are important sources of stress for piglets [44].

In the present study, the highest mean HCC was observed in the second breeding phase in winter, according to previous studies in pigs [45,46]. Also, López-Arjona et al. [47] did not observe a significant hair cortisol increase under heat stress conditions in pigs.

Interestingly, although previous studies suggest that darker-coated animals may have higher baseline HCC [25], in our study, the black-coated NL pigs consistently showed lower HCC values than the light-coated CH pigs at all time points and in both cycles.

Moreover, it was observed that the HCC levels in all the pigs markedly decreased from weaning to slaughter in both phases, in particular in the second phase for the NL pigs, indicating a different physiological response by the NL breed, which refers to a different NL animal response towards environmental and climatic fluctuations in the present study. However, other studies are needed to evaluate hair cortisol levels in this newly established breed to better understand HCC dynamics during pigs’ lives on conventional farms.

The NL pigs showed slower growth performance in the first phase, with a high THI. In the R2 phase, the NL pigs showed a higher backfat thickness, which is usually associated with a good nutritional efficiency, in the second cycle when fattening was between January and the end of June, with moderate values of the THI for heat stress, showing a better climatic attitude for cold climate, as stated previously, regarding NL weaners and growers. In fact, it is reported that local pig breeds are better adapted to specific environmental conditions and feeding resources and can more efficiently store fat than commercial breeds, representing a valuable genetic resource [48]. In general, both breeds exhibited a noticeable resilience for barren environments and resilience towards climatic fluctuations.

## 5. Conclusions

This study highlights the importance of an environmental microclimate in conventional pig farming without adequate insulation and automated climate control systems. The THI proved useful for assessing the thermal discomfort, which is increasingly relevant under changing climatic conditions. This preliminary study of NL pigs showed that this breed had growth performance comparable with a commercial breed, even if the local breeds usually show a slower growth rate. The two genotypes exhibited distinct physiological responses in the same environment. The CH pigs had higher hair cortisol concentrations than the NL pigs throughout all production phases, probably indicating different physiological patterns and different responses to environment and farming. In this study, the lower cortisol levels in the NL than CH pigs in the R2 cycle were linked to an increase in the backfat thickness, with moderate values of the THI. Although not a strictly native breed, Nero di Lomellina pigs could represent a trade-off between rusticity and a suitable production performance, demonstrating a good adaptability under barren farming conditions.

In conclusion, this preliminary data suggests that Nero di Lomellina pigs could be a valuable resource for low-input farming systems with limited environmental control thanks to their stable physiological profile and greater resilience. Further studies are needed to confirm the present data and also consider the influence of an outdoor farming system on the physiological status and productivity of this local breed.

## Figures and Tables

**Table 1:** Timelines of the two monitored rearing cycles.

Rearing Cycle	Farrowing Room	Post-Weaning	Growing	Fattening
R1	December	January	February–March	April–October
R2	September	October	November– December	January–June

**Table 2:** Reference values for critical lower and upper temperature limits and RH. THI values were calculated according to Equation (1).

Classes Based on LW	Weight Range (kg)	Critical Lower T (°C)	Critical Upper T (°C)	THI
0	Newborn	32	35	75–81
1	0–8	25	35	62–80
2	9–20	22	28	57–68
3	20–40	19	26	51–64
4	60	18	24	49–63
5	80	17	23	48–61
6	100	16	22	46–60
7	120	15	21	44–59

**Table 3:** Average body weight (±SEM) recorded for the two production cycles (R1 and R2) in each production phase.

	R1		R2	
	NL	CH	NL	CH
Weight, kg				
Birth	1.01 ± 0.006	0.97 ± 0.014	1.39 ± 0.022	1.38 ± 0.036
Weaning	8.12 ± 0.19 ^a^	9.17 ± 0.27 ^b^	8.64 ± 0.24	9.52 ± 0.52
Growing phase	57.85 ± 2.32 ^a^	66.71 ± 2.30 ^b^	56.0 ± 4.39	56.28 ± 2.90
Fattening phase	174.0 ± 4.46 ^a^	179.85 ± 1.80 ^b^	171.3 ± 1.23	167.3 ± 1.89
Backfat thickness at P2, mm				
Growing phase	6.47 ± 0.30	6.52 ± 0.24	6.30 ± 0.41	5.88 ± 0.23
Finishing phase	16.34 ± 1.41	18.74 ± 0.58	22.81 ± 0.97 ^A^	18.37 ± 0.10 ^B^

n = 14; significance level is intended for raw data, ^A, B^ *p* < 0.001, ^a, b^ *p* < 0.05.

**Table 4:** Modified Gompertz curve parameters (±SEM) for the two breeds in the two periods, predicting the weight at 500 d of age (^A, B^ *p* < 0.01; ^a, b^ *p* < 0.05).

Item	R1 CH	R2 CH	R1 NL	R2 NL
a	231.9 ± 8.2178 ^A^	237.6 ± 15.9525 ^A^	237.6 ± 15.141 ^B^	254.8 ± 21.7049 ^B^
b	0.0119 ± 0.00247	0.0107 ± 0.00326	0.0107 ± 0.00293	0.0121 ± 0.00301
c	0.9905 ± 0.00057	0.9911 ± 0.00087 ^a^	0.9911± 0.00073	0.992 ± 0.00085 ^b^

**Table 5:** Air pollutant concentrations (mg/m^3^) measured in R1 and R2 production phases.

Unit		Air Pollutant Concentrations (mg/m^3^)							
		R1				R2			
		NH_3_	CO_2_	N_2_O	CH_4_	NH_3_	CO_2_	N_2_O	CH_4_
Farrowing room	Mean	13.04	4191	1.85	49.70	6.2	2070.4	1.5	46.2
	Std. dev	0.56	152	0.55	2.47	0.9	208.8	0.5	6.8
Post-weaning	Mean	5.37	2858.93	1.71	18.39	6	2927	1.5	16.7
	Std. dev	0.18	110.11	0.34	1.82	2.11	179	0.15	4.11
Fattening	Mean	8.48	2554	0.61	28.60	4.9	2580	1.04	16.8
	Std. dev	4.05	1260	0.09	12.17	1.9	418	0.3	5.7

**Table 6:** THI mean values calculated in the two rearing cycles up to the growing phase, with mean (minimum–maximum).

	Farrowing	Post-Weaning	Growing
R1	62 (57–75)	64 (60–77)	59 (48–75)
R2	65 (60–81)	67 (57–82)	60 (54–74)

## Data Availability

Data is contained within the article.

## Abbreviations

The following abbreviations are used in this manuscript:

NL	Nero di Lomellina
CH	Commercial hybrid
HCC	Hair cortisol concentration
NH_3_	Ammonia
CO_2_	Carbon dioxide
N_2_O	Nitrous oxide
CH_4_	Methane
H_2_S	Hydrogen sulfide
TNZ	Thermoneutral zone
THI	Temperature–humidity index
RH	Relative humidity
T	Temperature
LW	Live weight
